# The Effect of Acute Continuous Hypoxia on Triglyceride Levels in Constantly Fed Healthy Men

**DOI:** 10.3389/fphys.2019.00752

**Published:** 2019-06-18

**Authors:** Jean-François Mauger, Étienne Chassé, Bimit Mahat, Clare Lindon, Nicolas Bordenave, Pascal Imbeault

**Affiliations:** ^1^School of Human Kinetics, Faculty of Health Sciences, University of Ottawa, Ottawa, ON, Canada; ^2^School of Nutrition Sciences, Faculty of Health Sciences, University of Ottawa, Ottawa, ON, Canada; ^3^Institut du Savoir Montfort, Hôpital Montfort, Ottawa, ON, Canada

**Keywords:** triglycerides, lipoproteins, prandial, lipase activity, normobaric hypoxia

## Abstract

**Introduction:**

Elevated plasma triglyceride (TG) concentrations are an important contributor to deleterious metabolic alterations. Evidence in animals suggest that acute exposure to an environment with reduced oxygen inhibits plasma TG clearance and causes important rise in plasma TG, especially in the postprandial state. The objective of this study was to characterize the effects of an acute exposure to normobaric hypoxia on prandial TG levels in 2 distinct lipoprotein subtypes in healthy humans: chylomicrons which are secreted by the intestine and carry dietary lipids, and denser TG carriers (mainly VLDL), which are secreted by the liver and carry endogenous lipids. Plasma lipolytic activity was also assessed. It was hypothesized that hypoxia would reduce prandial plasma lipolytic activity and raise prandial TG levels in both lipoprotein subtypes.

**Methods:**

Using a randomized crossover design, 9 healthy young men were studied for 6 h in a constantly fed state while being exposed to either normobaric hypoxia (FiO_2_ = 0.12) and normoxic conditions on two different days. Prandial glucose, TG, non-esterified fatty acid (NEFA), and post-heparin plasma lipolytic activity were measured during each session.

**Results:**

Six hours of exposure to hypoxia marginally increase prandial glycemia (+5%, *p* = 0.06) while increasing insulinemia by 40% (*p* = 0.04). Hypoxia induced a 30% rise in prandial NEFA levels and tended to slightly increased total prandial TG levels by 15% (*p* = 0.11). No difference was observed in TG concentrations and metabolism of chylomicrons between conditions. However, TG in the VLDL containing fraction decreased significantly overtime under normoxia but not under hypoxia (time × condition interaction, *p* = 0.02). No difference was observed in post-heparin plasmatic lipolytic activity between conditions.

**Conclusion:**

Acute hypoxia in healthy men tends to increase prandial VLDL-TG levels. These results lend support to the increased blood lipid levels reported in animals exposed acutely to lower partial pressures of oxygen.

## Introduction

Dietary triglyceride (TG) clearance has been reported to be impaired by acute hypoxia, causing an important rise in postprandial lipemia (Jun et al., 2012), a well-known risk factor for metabolic disorders such as cardiovascular diseases and type 2 diabetes (Zilversmit, 1995; Lewis et al., 2002). Subsequent studies showed, however, that the effects of acute hypoxia on lipemia were less obvious in fasting animals and practically absent when animals were studied under thermoneutral conditions (Muratsubaki et al., 2003; Jun et al., 2013). Nonetheless, 6-h of hypoxia (10% O2) at thermoneutrality, despite having no apparent effects on postprandial plasma TG concentrations, still induced an 80% reduction in the activity of white adipose tissue lipoprotein lipase (LPL) (Jun et al., 2013), the purported chief enzyme responsible for the clearance of TG-rich lipoproteins (TRL) (Wang and Eckel, 2009; Kersten, 2014).

In humans, the physiological effects of hypoxia have been studied throughout the last 50 years and multiple investigators reported plasma TG responses of individuals exposed to high altitude (>3500 m) or low oxygenation (Whitten and Janoski, 1969; Férézou et al., 1988, 1993; Young et al., 1989; Farias et al., 2006). However, control for critical confounders likely to alter plasma TG, such as physical activity, diet, cold exposure and weight loss/gain was generally lacking, which might explain the widely conflicting observations regarding the effects of hypoxia on plasma TG levels reported in those studies. Recently, we reported that 6 h of intermittent normobaric hypoxia at rest had no effect on plasma TG concentrations following the consumption of an isocaloric fat-rich liquid meal (Mahat et al., 2016). Because we thought that the postprandial rise in insulin might have inhibited adipose tissue lipolysis and suppressed hepatic very-low density lipoprotein (VLDL) production, we conducted another study in the fasting state under continuous (i.e., not intermittent) hypoxic conditions. Again, we observed no effect of hypoxia on circulating TG (Mahat et al., 2018). Interestingly, despite no changes in plasma TG concentrations, our previous studies reported significant increases (∼+35%) in circulating non-esterified fatty acids, which is an important contributor to hepatic lipogenesis and very-low density lipoprotein production (Nielsen and Karpe, 2012).

One limitation of our previous studies was that they rely solely on plasma TG concentrations, which provide limited information regarding TG and lipoprotein dynamics. To get further insight into lipoprotein metabolism under hypoxic conditions, we constantly fed young healthy males to induce a steady prandial state and used retinol to study the dynamics of buoyant intestinal born chylomicrons. We hypothesized that hypoxia would disturb chylomicron metabolism by decreasing prandial plasma lipolytic activity and hence reduce chylomicron clearance.

## Materials and Methods

### Subjects

Healthy young men (*n* = 9) were recruited from the University of Ottawa population. Study subjects provided written consent and the study protocol was approved by the Research and Ethics Board of the University of Ottawa. Exclusion criteria included: weight fluctuations, hypertension, cardiovascular diseases, diabetes, habitual sleep duration of less than 7 h per night, lactose intolerance, and current smoking status.

### Anthropometric and Metabolic Measurements

Body weight was determined with a standard beam scale (HR-100, BWB-800AS; Tanita, Arlington Heights, IL, United States) after urination and height was measured using a standard stadiometer (Perspective Enterprises, Portage, MI, United States). The percentage of fat mass (%FM), total fat mass (FM) and fat free mass (FFM) were measured using dual energy X-ray absorptiometry (DXA) (General Electric Lunar Prodigy, Madison, Wisconsin; software version 6.10.019). Resting energy expenditure (REE) was measured in a thermoneutral dark room for 30 min using a Vmax Encore 29 System metabolic cart (VIASYS Healthcare Inc., Yorba Linda, CA, United States) following a 12 h overnight fast. Daily energy expenditure (DEE) on experiment days was estimated by multiplying the participants REE by a physical activity factor of 1.375 (Harris and Benedict, 1918).

### Experimental Protocol and Procedures

This study was a randomized crossover study consisting of two experimental sessions. Prior to each experimental session, volunteers were counseled to sleep at least 7 h per night, to restrain from any exercises, caffeine and alcohol for at least 36 h, and to consume a provided standardized evening dinner between 7:00 and 8:00 PM (680 kcal; 54% from carbohydrates, 22% from fat, and 24% from protein). On study days, volunteers presented themselves at the laboratory at 7:30 AM after a 12-h overnight fast. Participants were allowed only to drink water. Weight and blood pressure measurements were performed after urination but before an intravenous line was inserted in the antecubital vein for blood sampling. Volunteers were thereafter asked to consume the first of twelve liquid meals (35% of calories from fat, 55% from carbohydrates, and 10% from protein), providing 40% of their estimated DEE. Participants were then exposed to either hypoxia (FIO_2_ = 0.12) or ambient air (normoxia) for 6 h. Liquid meal servings were provided every 30 min and triglycerides reached a steady state after 180 min on average. Volunteers remained in a semi-recumbent position and occupied themselves by watching television. Conversation with the evaluators was limited. Sleep was not allowed. Blood (6 ml) was collected every hour following standard procedures. At time 180 min, participants were asked to ingest 60000 IU of retinyl-palmitate in the form of 6 gelatin capsules to study chylomicron metabolism. A heparin injection (Sandoz Canada Inc., QC, Canada) at a dose of 60 units per kg of body weight was given after completion of the 6-h experimental protocol and a last blood sample was drawn 20 min after to analyze plasma lipolytic activity.

Pulse rate (HR) and oxyhemoglobin saturation (SpO_2_) were recorded every 2 s during both experimental sessions using a Masimo, radical 7 unit (Masimo, Irvine, CA, United States). A mean average was calculated with all values for each experimental session. Blood pressure (BP) was measured following a 5–10 min rest period after the arrival of the participant to the laboratory, at mid-experiment (T180) and at the end of the experimental session (T360) with an automatic sphygmomanometer (American Diagnostic Corporation, E-sphyg 2, Hauppauge, NY, United States) following the Canadian Society of Exercise Physiology (CSEP) standard procedures (CSEP, 2013). Mean arterial pressure (MAP) was calculated with the following formula: MAP = (1/3^∗^systolic pressure) + (2/3^∗^diastolic pressure).

### Energy Expenditure and Fuel Utilization


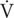
O_2_ and production of 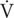
CO_2_ were measured hourly for 30 min using a calibrated Vmax Encore 29 System metabolic cart (VIASYS Healthcare Inc., Yorba Linda, CA, United States). 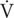
O_2_ and 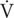
CO_2_ are expressed in STPD. Total carbohydrate (Rcho_ox_) and lipid (Rfat_ox_) oxidation rates (g/min) were calculated as described previously (Livesey and Elia, 1988, AJCN), with the following formulas:

$${\mathrm{RCHO}}_{\text{ox}}\;(g/\min)=4.59{\;\mathrm{VCO}}_{2}\;(L/\min)-3.23{\;\mathrm{VO}}_{2}\;(L/\min)$$

$${\mathrm{Rfat}}_{\text{ox}}\;(g/\min)=-1.70{\;\mathrm{VCO}}_{2}\;(L/\min)+1.70{\;\mathrm{VO}}_{2}\;(L/\min)$$

Rpro_ox_ was set at 20% since participants were fed with a mixed meal (Labayen et al., 1999). Energy expenditure was calculated using the following energy conversion factors: CHO 16.3 KJ/g, lipids 40.8 KJ/g, and proteins 19.7 KJ/g (Péronnet and Massicotte, 1991).

### Normobaric Hypoxic Exposure

Each of the two experimental sessions was performed in a climate-controlled chamber (volume of approximately 64 m^3^). During normoxia sessions, only ambient air was used. During hypoxia sessions, five O_2_ extractors (CAT12 oxygen extractor, Altitude Control Technologies, Lafayette, CO, United States), connected to the chamber kept FIO_2_ levels stable at 0.1200. Temperature fluctuated between 27°C and 29°C during the experimental sessions and relative humidity remained stable at approx. 45%. Hypoxia was generally well tolerated and presented no adverse effects apart from mild headaches in four participants.

### Plasma Analyses

Plasma was obtained by centrifugation at 3200 rpm for 12 min at 4°C immediately after blood collection. Commercially available colorimetric enzymatic assays were used to measure plasma triglycerides, glucose, and non-esterified fatty acid (NEFA) levels (Wako Chemicals USA Inc., VA, United States) as previously described (Imbeault et al., 2009). Denser lipoprotein TG concentration was calculated by subtracting chylomicrons TG concentrations from total plasma TG concentrations. Plasma insulin was assessed using a commercial enzyme-linked immunosorbent assay (Millipore EMD, Burlington, MA, United States). Plasma lipolytic activity was measured on post-heparin plasma using the Enzchek lipase substrate, a triglyceride analog which becomes fluorescent upon hydrolysis (Basu et al., 2011). Substrate hydrolysis was monitored for 20 min and maximal velocity was calculated using the Gen 5 software (BioTek, Winooski, VT, United States).

### Chylomicrons Isolation

For eight participants, chylomicrons were isolated using a modified method from Calsake and Packard (Rifai et al., 2000). Briefly, 1 ml of prandial plasma was overlayed with 0.5 ml of deionized water and centrifuged for 40 min at 12000 rpm (20000 RCF at maximal radius) at 4°C using a Thermo Fisher Scientific 30 × 2 fixed-angle sealed rotor (75003652) in a Sorvall ST16R centrifuge. Infranatant (1 ml) was discarded, 0.5 ml of sterile physiological saline was added to the supernatant and the procedure was repeated 2 times for a total of 3 centrifugations. Chylomicrons could not be isolated from 1 participant because of insufficient plasma volume at most time points.

### Retinoid Analysis

Retinoïds were extracted using the method described by Kim and Quadro (2010) and quantified by reverse-phase HPLC from isolated chylomicrons. Briefly, 100 μL of an 1 μg.μL^−1^ retinyl acetate internal standard was added to 500 μL of each sample of isolated chylomicrons. The samples were then extracted with 4 ml hexane. Hexane fraction was recovered by centrifugation and extracted with 500 μL deionized water. After recovery, the hexane fraction was dried under vacuum at room temperature, reconstituted with 200 μL HPLC mobile phase and injected into the HPLC system.

The HPLC system was equipped with a Waters Spherisorb 5 μm ODS2 4.6 × 250 mm analytical column (Waters, Millford, MA, United States) and a Gilson UV/Vis-155 detector set at 325 nm (Gilson, Middleton, WI, United States). The mobile phase consisted in acetonitrile, methanol and methlyenechloride (70 : 15 : 15 v/v) at a flow rate of 1.8 ml.min^−1^. Based on the internal standard quantification, recovery was >98%. Retinol and retinyl palmitate were quantified from calibration curves that exhibited the same linear correlation factors of *R*^2^ = 0.997. Their respective detection limits were determined to be 1.95 and 3.91 ng per injection.

### Statistical Analysis

**FIGURE 1:**
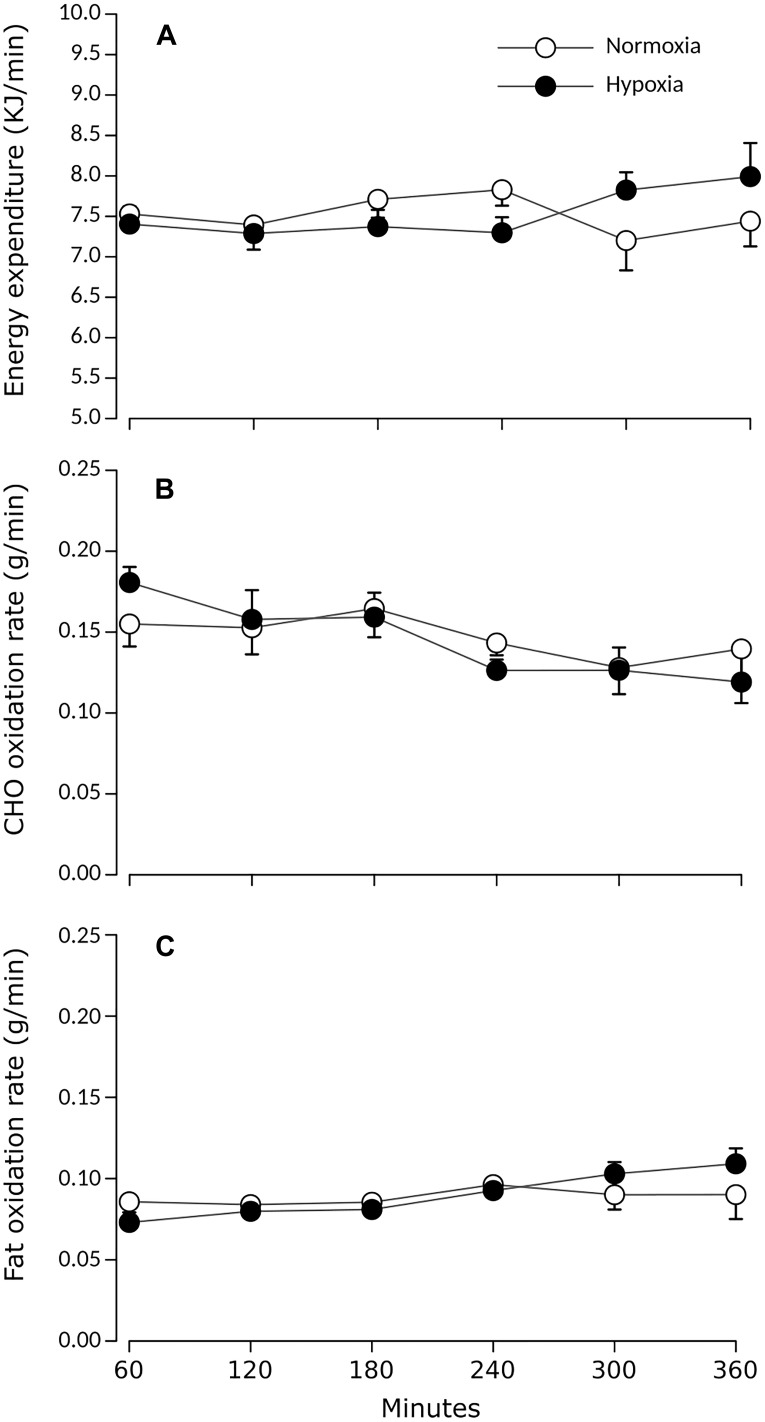
Prandial **(A)** energy expenditure, **(B)** carbohydrate, and **(C)** lipid oxidation rates during normoxia and continuous normobaric hypoxia in constantly fed men. Values are means ± standard errors adjusted for between-subjects variability.

**FIGURE 2:**
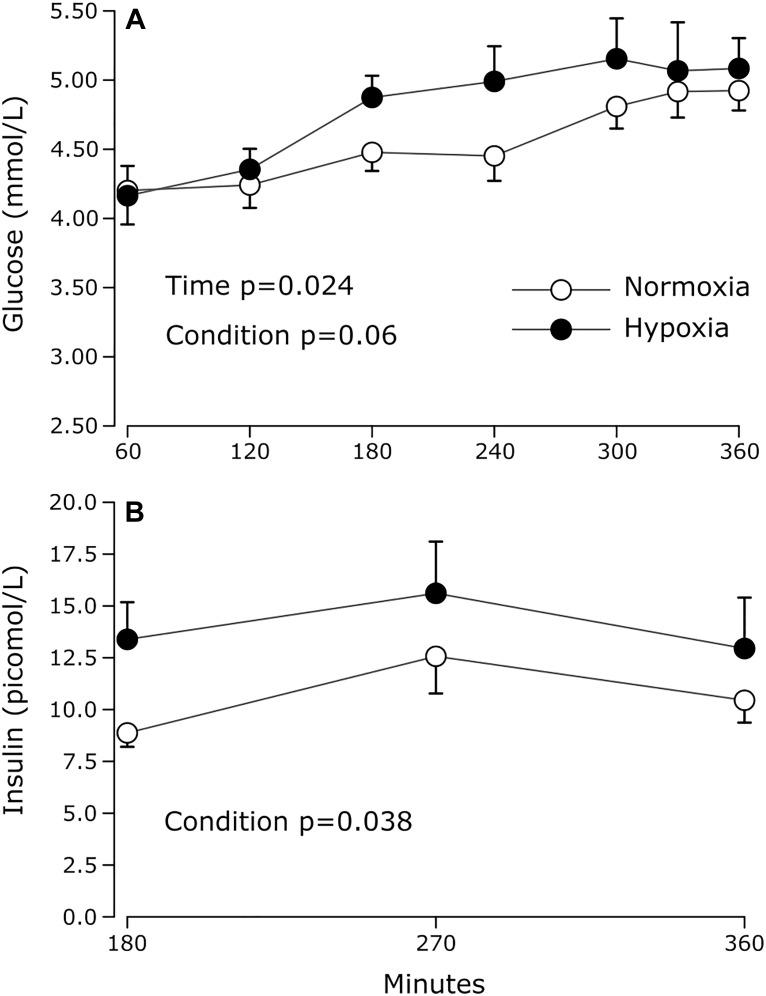
Prandial plasma **(A)** glucose and **(B)** insulin levels measured during normoxia and continuous normobaric hypoxia in constantly fed men. Values are means ± standard errors adjusted for between-subjects variability.

**FIGURE 3:**
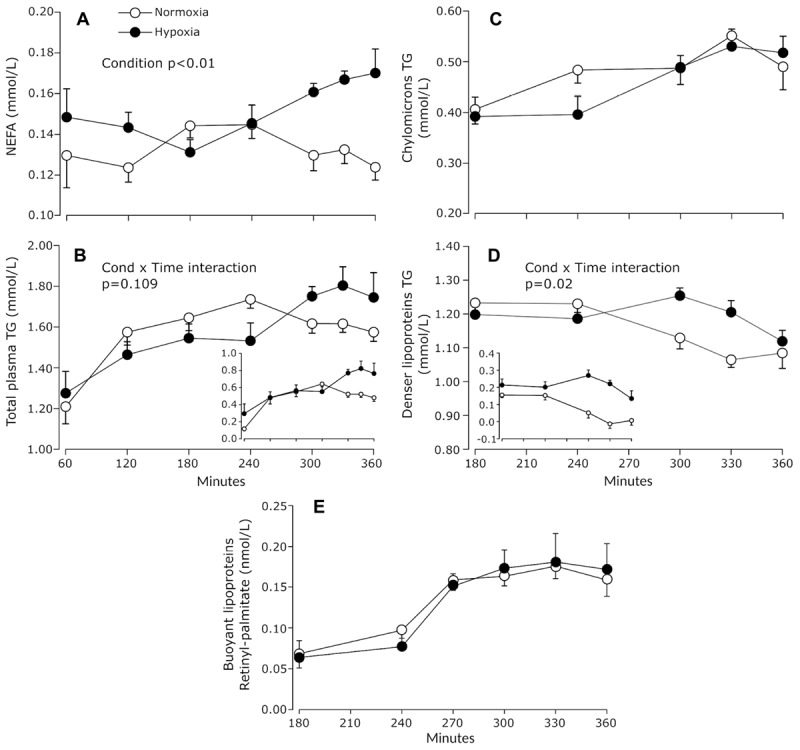
Prandial plasma NEFA **(A)**, total TG **(B)**, chylomicrons TG **(C)**, and denser lipoprotein TG **(D)** measured during normoxia and continuous normobaric hypoxia in constantly fed men. Inserts in panels **(B,D)** show baseline (fasting) adjusted values. Panel **(E)** shows the concentrations of retinyl-palmitate in chylomicrons. Values are means ± standard errors adjusted for between-subjects variability.

Statistical analyses were conducted using the Jamovi statistical software (Version 0.9 ^1^, RRID:SCR_016142). Analysis of variance (ANOVA) with repeated measures were performed with condition and time as within subject independent variables. The Greenhouse-Geiser correction was used when the sphericity assumption was violated according to Mauchly’s test of sphericity. Log transformation was used to analyze insulin and retinyl-palmitate because these variables were widely dispersed. Alpha was set at 0.05. Separate *post hoc* one factor ANOVA were conducted with time and condition as main effect to interpret time × condition interactions when statistically significant. Errors bars in Figure 1–3 were adjusted to account for between-subjects variability, which better reflects the statistical power of the study crossover design (Cousineau, 2005).

## Results

### Characteristics of Participants

The study sample consisted of nine healthy men whose characteristics are summarized in Table 1. Participants’ weights were stable (±0.24 kg) between both experimental sessions, which were separated by 7 ± 4 days on average.

**Table 1:** Characteristics of participants (*n* = 9).

Age (y)	24.4 ± 4.5	
Height (cm)	178.9 ± 3.6	
Weight (kg)	77.8 ± 8.0	
Body mass index (kg/m^2^)	24.3 ± 2.6	
Lean Mass (kg)	65.9 ± 5.7	
Fat Mass (kg)	8.8 ± 3.7	
Fat Mass (%)	11.5 ± 3.8	
**Fasting plasma parameters**	**Normoxia visit**	**Hypoxia visit**
Triglyceride (mmol/L)	96.8 ± 23.8	86.8 ± 16.8
Non-esterified fatty acids (mmol/L)	0.475 ± 0.223	0.313 ± 0.170
Glucose (mmol/L)	4.40 ± 0.18	4.61 ± 0.29
Insulin (picomol/L)	5.19 ± 4.72	3.59 ± 2.54

*Data are expressed as mean ± standard deviation.*

### Cardiorespiratory Responses

Table 2 displays variations in heart rate, blood pressure and oxyhemoglobin saturation during both experimental sessions. During hypoxia, mean heart rate was 20% higher (+13 bpm) (*p* < 0.001) while mean oxyhemoglobin saturation levels were 15% lower (*p* < 0.001) as compared to values measured during normoxia. Neither MAP, systolic pressure, nor diastolic pressure differed between experimental conditions.

**Table 2:** Mean heart rate, blood pressure, and oxyhemoglobin saturation during normoxia or continuous normobaric hypoxia in constantly fed men.

	Normoxia	Hypoxia
Heart rate (bpm)	62.9 ± 9.3	76.3 ± 10.4^∗^
Systolic blood pressure (mmHg)	119 ± 6	117 ± 6
Diastolic blood pressure (mmHg)	69 ± 8	72 ± 5
Mean arterial pressure (mmHg)	86 ± 6	87 ± 5
SpO_2_ (%)	97.5 ± 1.3	82.4 ± 2.7^∗^

*Data are expressed as means ± standard deviation. ^∗^Significantly different compared to normoxia, p < 0.001.*

### Substrate Oxidation

Figure 1 presents prandial energy expenditure and substrate utilization rates for each condition. Total energy expenditure remained stable over time and did not differ between conditions. Rcho_ox_ and Rfat_ox_ did not differ between conditions (main effect of condition *p* > 0.05) and did not significantly change over time (main effect of time *p* > 0.05).

### Plasma Glucose and Insulinemia

The effects of hypoxia on plasma glucose and insulin concentrations are summarized in Figure 2. Prandial blood glucose levels increased significantly over time in both conditions (main effect of time *p* = 0.02). After 180 min of exposure, glucose levels remained stable over time in both conditions (main effect of time after 180 min, *p* = 0.392). During this period, average glucose levels were marginally but statistically higher under hypoxia by 7% (main effect of condition *p* = 0.03). Prandial insulinemia was significantly higher by 51% on average during hypoxia (main effect of condition *p* = 0.04), with increases observed in 7 out of 9 subjects, 6 of which showed increases greater than 25%.

### Plasma Lipid Parameters

As shown in Figure 3, prandial NEFA levels were significantly increased under hypoxia compared to normoxia (main effect of condition *p* < 0.01). In the last hour of exposure, NEFA concentrations were 30% greater under hypoxia compared to normoxia. Increase in NEFA was observed in 8 out of 9 subjects ranging from 7 to 95% and increases greater than 25% were observed in 6 subjects out of 9. Only one subject showed lower NEFA concentrations during hypoxia compared to normoxia (−10.0% decrease).

Prandial TG concentrations rose in a significant manner in response to meals in both experimental conditions and the increase tended to be greater under hypoxia (condition × time interaction *p* = 0.109) (Figure 3B). On average, plasma TG concentrations were 15.5% ± 28.8% higher in the last hour of hypoxia compared to normoxia. When fasting TG concentrations were subtracted from prandial TG concentrations, plasma TG concentration averaged for the last 60 min were 112% higher under hypoxia, with 6 subjects out of 9 showing increase in the range of 91 to 300% (main effect of condition *p* = 0.071).

In eight subjects, triglycerides were assessed in two distinct lipoprotein pools: buoyant TG-rich lipoproteins, comprising mostly chylomicrons, and denser lipoproteins such as chylomicron remnants and VLDL. Triglyceride concentrations in chylomicrons increased slightly over time in both conditions (main effect of time *p* = 0.035) (Figure 3C). Triglycerides associated with denser lipoproteins (VLDL and remnants) decreased slightly but significantly in the last 3 h of normoxia exposure, while no such reduction was observed under hypoxia (condition × time interaction *p* = 0.02) (Figure 3D). As a result, average TG concentrations associated with the denser lipoprotein fraction tended to be 16% higher in the last hour of hypoxia (main effect of condition *p* = 0.10).

To investigate the effect of hypoxia on chylomicrons metabolism, participants were given an oral bolus of 60000 IU of retinyl-palmitate at the 180 min time point, at which point prandial triglycerides had reached steady-state. Retinyl-palmitate is incorporated into chylomicrons within enterocytes and most (70–75%) of the chylomicron retinyl-palmitate remains with the lipoprotein until chylomicron remnants are internalized in the liver (Paik et al., 2004). Therefore, retinyl-palmitate can be used to follow the appearance and clearance of chylomicrons from the circulation. As shown in Figure 3E, hypoxia did not affect the dynamics of retinyl-palmitate within the buoyant lipoprotein fraction, which suggests that hypoxia affected neither the intestinal production nor the vascular catabolism of these lipoproteins.

Post-exposition heparinized plasma lipolytic activity was not significantly different between conditions (normoxia 1.27 × 10^6^ ± 0.08 × 10^6^ RFU/min vs. hypoxia 1.24 × 10^6^ ± 0.11 × 10^6^ RFU/min, paired *T*-test *p* = 0.62, data not shown).

## Discussion

This study is, to our knowledge, the first to examine the effects of continuous normobaric hypoxia on lipid metabolism under prandial steady state conditions. We report that a 6-h exposure to hypoxia tends to increase total prandial TG levels by 15% (0.16 mmol, *p* = 0.11). Based on indirect calorimetry, the rise in prandial TG levels under hypoxia appears not to be the result of alterations in intestinal lipid absorption, energy expenditure nor reduction in lipid oxidation rate, but rather by hypoxia blunting the prandial decrease in TG content of denser TG-rich lipoprotein observed in normoxic conditions. Together, these results partially support our hypothesis that acute hypoxia negatively impacts prandial TG metabolism.

We show that plasma NEFA, glucose and insulin levels are all significantly elevated under hypoxic conditions. In healthy individuals, postprandial insulin secretion prevents excessive increases in plasma glucose levels, but also strongly suppresses adipose tissue lipolysis, so that plasma NEFA levels drop drastically after food intake. Intermittent or constant hypoxia exposure has been shown to induce a rise in glucose levels (Newhouse et al., 2017) and/or transitory insulin resistance (Louis and Punjabi, 2009; Peltonen et al., 2012). Peltonen et al. demonstrated that sympathetic activation may be partly responsible for this effect (Peltonen et al., 2012). In the present study, the significant rise in HR observed under hypoxia suggests that sympathetic activation effectively occurred under hypoxia, which could explain the higher plasma NEFA, glucose and insulin levels observed under these conditions.

Total plasma TG levels increased over time in both experimental conditions and, compared to normoxia, tended to be 15% greater after 6 h of normobaric hypoxia (Figure 3). Fractionation of TG-rich lipoproteins into chylomicrons and denser subclasses showed that this trend toward elevated total plasma TG was paralleled by a statistically significant 16% increase in the TG content of the denser lipoprotein pool. The method we used to fractionate plasma lipoproteins yield a buoyant fraction (*d* < 1 g/ml) likely highly enriched in intestinal born chylomicrons and a denser fraction (*d* > 1 g/ml) likely mainly composed of chylomicron remnants and liver born VLDL. The TG content of a lipoprotein fraction reflects the equilibrium between the appearance and clearance rate of its TG moiety. It has already been shown that dietary lipid absorption is not affected by altitude up to 5 500 m (Kayser and Verges, 2013). Thus, assuming no changes in the appearance rate of chylomicrons-associated TG under hypoxia, the lack of difference in both the TG content and retinyl-palmitate dynamics in the buoyant lipoprotein pool suggests that chylomicron TG clearance might as well not have been altered by hypoxia. This hypothesis is in line with our observation that plasma post-heparin lipolytic activity is not affected by acute hypoxia as well as with previous observations from our group showing that acute intermittent hypoxia does not seem to affect postprandial subcutaneous abdominal adipose tissue LPL activity (Mahat et al., 2016). Consequently, the apparent trend toward higher plasma TG under hypoxia may rather be explained by changes in the metabolism of denser TG carrying lipoproteins such as VLDL and chylomicron remnants. Based on our observation that hypoxia does not seem to affect chylomicron TG clearance, it can then be hypothesized that postprandial VLDL-TG clearance may as well not be affected by hypoxia, since both lipoprotein subtypes follow the same vascular degradation pathway involving LPL. If this assumption is true, then the hypoxia-induced increase in denser lipoprotein TG content could be attributed to either (1) a decreased tissue uptake of VLDL remnants and chylomicron remnants, or (2) an increased output of VLDL from the liver as a way to cope with the increase lipid influx under postprandial hypoxic conditions. While the regulation of VLDL and chylomicron remnants uptake is still poorly understood (Ramasamy, 2013), VLDL production is generally accepted to be reduced in the postprandial state, mainly because insulin inhibits adipose tissue lipolysis, thus reducing hepatic NEFA delivery, the main substrate for VLDL-TG production (Nielsen and Karpe, 2012). In our previous study conducted in the fasted state, the increase in circulating NEFA under hypoxia was not matched by an increase in plasma TG concentration, suggesting that in the fasted state, increased availability of NEFA alone may not acutely affect hepatic VLDL production (Mahat et al., 2018). In the present study, however, it could be suspected that the combination of chylomicron remnants delivery and elevated plasma NEFA may have provided enough lipid substrate to drive an increase in hepatic VLDL production. Consistent with this hypothesis, a recent report suggests that journey at altitude may increase the liver output of TG constituted of adipose tissue lipolytic products such as palmitic and oleic acid, supporting the concept that hypoxia may promote lipid recycling and stimulate hepatic TG secretion (O’Brien et al., 2019).

### Strengths and Weaknesses

The main weaknesses of the present study regard the size and composition of its sample. The relatively small sample size was, however, counterbalanced by the statistical strength of the crossover design so that significant changes in the TG content of denser lipoproteins were detected. Our sample consisted exclusively of healthy young men, which prevents extending our conclusions to other individuals, such as women or individuals with excessive adipose tissue levels, the later groups being particularly at risk of suffering from the deleterious metabolic effects of hypoxia in the form of obstructive sleep apnea (Resta et al., 2001; Peppard et al., 2013).

## Conclusion

The present work partially supports previous observations from animal studies showing that acute normobaric hypoxia may negatively affect postprandial triglyceride levels. Our results suggest that hypoxia-induced adverse alterations in prandial TG levels are not caused by a decrease in lipid oxidation nor an impaired vascular catabolism of chylomicron TG, but rather seems to be associated with changes in the metabolism of denser TG containing lipoprotein, possibly chylomicron remnants and VLDL. Future studies are required to better our understanding of the regulatory cascades leading to changes in vascular lipid homeostasis under hypoxic conditions such as high-altitude exposure as well as pathological conditions of oxygen deprivation such as obstructive sleep apnea or chronic obstructive pulmonary disease.

## Data Availability

The datasets generated for this study are available on request to the corresponding author.

## Ethics Statement

This study was carried out in accordance with the recommendations of the Research and Ethics Board of the University of Ottawa with written informed consent from all subjects. All subjects gave written informed consent in accordance with the Declaration of Helsinki. The protocol was approved by the Research and Ethics Board of the University of Ottawa.

## Author Contributions

ÉC, JF-M, and PI conceived and designed the experiments. ÉC, CL, BM, and JF-M performed the experiments. JF-M, ÉC, BM, and NB analyzed the data. JF-M, ÉC, NB, and PI interpreted the data. All authors edited, revised, and approved the final version of the manuscript.

## Conflict of Interest Statement

The authors declare that the research was conducted in the absence of any commercial or financial relationships that could be construed as a potential conflict of interest.

## Footnotes

1 www.jamovi.org
